# The Georgia Medical Association

**Published:** 1882-03-20

**Authors:** 


### *THE GEORGIA MEDICAL ASSOCIATION*

Will meet the present year in Atlanta, on Wednesday, April 19. All necessary arrangements will be made for the proper reception of the Association by the local profession of the city. It is hoped there will be a full turn out from all sections of the State.

